# Trematodes of Genera *Gyrabascus* and *Parabascus* from Bats in European Russia: Morphology and Molecular Phylogeny

**DOI:** 10.3390/biology11060878

**Published:** 2022-06-08

**Authors:** Nadezhda Yu. Kirillova, Sergei V. Shchenkov, Alexander A. Kirillov, Alexander B. Ruchin

**Affiliations:** 1 Samara Federal Research Scientific Center RAS, Institute of Ecology of Volga River Basin RAS, Togliatti 445003, Russia; ievbras2005@mail.ru (N.Y.K.); parasitolog@yandex.ru (A.A.K.); 2 Department of Invertebrate Zoology, Saint Petersburg State University, St. Petersburg 199034, Russia; sergei.shchenkov@gmail.com; 3 Joint Directorate of the Mordovia State Nature Reserve, National Park “Smolny”, Saransk 430005, Russia

**Keywords:** Trematoda, *Gyrabascus*, *Parabascus*, 28S rRNA, molecular phylogeny, taxonomy, bats, Microchiroptera

## Abstract

**Simple Summary:**

The ecology of bats determines their unique parasitic fauna. Most species of worms from bats are highly specialized parasites. We studied parasitic worms of bats that died of natural causes, using classical morphological and molecular phylogenetic approaches. Original drawings, descriptions, and results of molecular phylogenetic analysis for five species of trematodes were provided. We established a broad morphological variability in the studied trematode species, which means that the identification of closely related species may be problematic for researchers. We proposed a taxonomic key for the reliable identification of the studied trematode species. The results of our study contribute to the knowledge of bat helminths and host-parasite relationships in general.

**Abstract:**

Morphological variability of trematodes from bats (Chiroptera) is poorly studied. Since the variability of adult digenean specimens may be rather high, morphological features are often insufficient for the identification of closely related species, and confirmation with the use of molecular data is required. The aim of our study was to combine the morphological and molecular phylogenetic analyses of several bat trematodes from the genera *Gyrabascus* and *Parabascus* (Pleurogenidae): *Gyrabascus amphoraeformis*, *Gyrabascus oppositus*, *Parabascus lepidotus*, *Parabascus duboisi*, and *Parabascus semisquamosus*, of which *G. amphoraeformis* and *G. oppositus* are little known in European Russia. We made detailed morphological descriptions of these trematodes from several definitive hosts, analyzed morphometric features, and generated new partial sequences of the 28S rRNA gene. A broad variability of trematodes of the genera *Gyrabascus* and *Parabascus* was revealed both from various host species and from specimens of the same host species. We propose a new taxonomic key for the identification of the studied species. Certain host specificity of these trematodes was revealed.

## 1. Introduction

Bats (Chiroptera) are the only mammals capable of active flight which strongly affects their food chains. Chiropterans harbor a unique helminth fauna, and the host specificity of their parasites is generally high [1,2].

Twenty-seven species of bats are found in European Russia and sixteen of them are known from the Volga Upland [3,4,5,6]. In recent decades, studies of bats in Russia have been devoted to various aspects of ecology, distribution, taxonomy, and parasite fauna [6,7,8,9,10,11,12,13,14].

Presently, 35 species of helminths are known for bats in the Middle Volga region, including 23 trematodes [15,16,17,18,19,20,21,22,23]. The study of trematodes, as well as of other parasitic worms, is necessary due to their epidemiological and epizootic importance. Some species of trematodes are known as causative agents of dangerous helminthiasis. The parasitological potential of the Middle Volga region is very high. In total, 34 species of trematodes of the 216 found in terrestrial vertebrates of the region may pose a serious threat to humans and wild and domesticated animals [16,17,18,19]. Yet bats, due to their high degree of ecological specialization and isolation, have not listed dangerous trematode species.

The variability of morphological features in trematodes from bats has seldom been studied. Skvortsov [24] noted that bat trematodes are characterized by a broad morphological variability, indicating the variability of a number of diagnostic morphological features, such as the location of the ovary, testes, cirrus sac, etc. Data on the morphological variability of *Plagiorchis* species from bats have been provided by Sharpilo and Tkach [25] and Tkach et al. [26]. Odening [27] has given measurements of *Parabascus duboisi* (Hurkova, 1961) from *M. daubentonii*, indicating a high variability of this parasite, as well as drawings of *P. duboisi* from various hosts with clearly visible differences in general morphology.

Zdzitowiecki [28] has provided detailed descriptions of *Gyrabascus* (former *Allassogonoporus*) *amphoraeformis* (Mödlinger, 1930), *Parabascus lepidotus* Looss, 1907, and *P. duboisi* from various bat species, showing the host-induced variability of these parasites. The morphological variability of bat trematodes of the genus *Parabascus* Looss, 1907 was discussed in the papers of Khotenovsky [29] and Sokolov et al. [30]. The variability of morphometric features of the trematode *Prosthodendrium ascidia* (Beneden, 1873) from various hosts is shown in Kirillov et al. [17].

Trematodes from the genera *Parabascus* and *Gyrabascus* Macy, 1935 are Holarctic species [29,31,32]. The distribution of most trematode species {*G. amphoraeformis*, *Gyrabascus oppositus* (Zdzitowiecki 1969), *P. duboisi*, *Parabascus semisquamosus* (Braun, 1900)} used in this study is limited to Europe. Only *P. lepidotus* has a Western Palearctic distribution [28,30,33].

*Parabascus semisquamosus* was described by Braun [34] from the bats in Germany as *Distomum semisquamosum*, *Parabascus lepidotus* was described by Looss [35] from a bat in Egypt, and *Parabascus duboisi* was found by Hurkova [36,37] in *M. daubentonii* in former Czechoslovakia (as *Parabascus* sp.).

*Gyrabascus amphoraeformis* was described by Modlinger [38] in Hungary from *Myotis blythii* Tomes, 1857 as *Distomum amphoraeformis*. Lotz and Font [31] synonymize the family Alassogonoporidae Skarbilovich, 1947 and the genus *Alassogonoporus* Olivier, 1938 with Gyrabascidae Macy, 1935 and *Gyrabascus*, respectively.

*Gyrabascus oppositus* was described as *Parabascus oppositus* by Zdzitowiecki [28] from bats in Poland. Earlier, Mituch [39] found *G. oppositus* in *Miniopterus schreibersii* Kuhl, 1817 in Hungary, but defined it as *P. semisquamosus.* Skvortsov [24] pointed out a significant similarity between the descriptions and drawings of *G. amphoraeformis* and *G. oppositus* given in Zdzitowiecki [28] and listed the latter species as a synonym of *G. amphoraeformis*. Khotenovsky [29] considers *G. oppositus* as a synonym for *P. lepidotus* without any explanation. A recent study of *G. oppositus* from *Pipistrellus kuhlii* (Kuhl, 1817) by Sokolov et al. [30] confirmed its validity and contributed to the revision of the generic affiliation of this species and its transfer to the genus *Gyrabascus*.

Tkach et al. [40,41] have repeatedly emphasized that morphological features are not always sufficient for the identification of trematode species and genera and that confirmation by molecular data is required. Only a few studies have been conducted on DNA sequencing of bat helminths [26,30,40,41,42,43,44].

Here, we studied five species of pleurogonid trematodes—*Gyrabascus amphoraeformis*, *Gyrabascus oppositus*, *Parabascus duboisi*, *Parabascus lepidotus*, and *Parabascus semisquamosus*—parasitizing bats in the Middle Volga region (Russia). The aim of our research was to provide reliable identification of these trematodes by combining morphological and morphometric analyses with the newly obtained molecular phylogenetic data.

## 2. Materials and Methods

### 2.1. Parasite Collection and Examination

Adult trematode specimens were collected from dead bats in 2019–2021 in two localities in the Republic of Mordovia (Russia): Pushta village (54°42′56″ N, 43°13′31″ E) and Smolny village (54°43′23″ N, 45°17′03″ E). No animals were killed intentionally for our research. Some dead specimens of bats were kindly provided by the staff of the Mordovia Nature Reserve and National Park “Smolny”. Several dead bats were provided by rural residents. Animals died of natural causes or were killed by domestic cats. Necropsy was performed on bats within approximately 1–9 h of their death. Only alive motile adult trematodes were collected for further investigations.

In total, we examined 16 bat specimens that belonged to six species: three *Myotis brandtii* Eversmann, 1845, one *Myotis daubentonii* Kuhl, 1817, five *Nyctalus noctula* Schreber, 1774, one *Nyctalus leisleri* (Kuhl, 1817), two *Pipistrellus nathusii* Keyserling et Blasius, 1839, and four *Vespertilio murinus* Linnaeus, 1758.

For the morphological examination, the trematodes were recovered from the intestine and killed by careful heating in water under identical conditions. The trematodes were stained with aceto-carmine, dehydrated in an ethanol series (70–96%), cleared in clove oil, and mounted in Canada balsam [45,46]. Trematode specimens for molecular phylogenetic analysis were fixed in 96% ethanol and stored at +4 °C.

In total, 109 specimens of trematodes were studied: 25 *Gyrabascus oppositus*, 1 *Gyrabascus amphoraeformis*, 28 *Parabascus duboisi*, 25 *Parabascus lepidotus*, and 30 *Parabascus semisquamosus*. Drawings were made using an MBI-9 light microscope with the Levenhuk M500 BASE Digital Camera and drawing tube RA-7. All the measurements are given in micrometers. For a comparative analysis of the morphology and measurements of trematodes, we used only those works that contained morphological drawings and morphometric data on the trematodes species of interest.

The taxonomic identification and morphological examination of the helminths were carried out in the Laboratory of Population Ecology of the Institute of Ecology of the Volga Basin of the Russian Academy of Sciences (Togliatti, Russia). The trematodes were identified according to Zdzietowiecki [28], Khotenovsky [29,47,48], Skvortsov [49], Sharpilo, Iskova [33], Odening [27], Kirillov et al. [17], and Sokolov et al. [30]. The voucher specimens of trematodes are stored in the parasitological collection of the Institute of Ecology of Volga Basin of RAS (IEVB RAS), a branch of the Samara Federal Research Center of the Russian Academy of Sciences.

The helminth taxonomy is given according to Lotz and Font [31,32], Fauna Europaea (https://fauna-eu.org/, accessed on 22 February 2022) [50], and Sokolov et al. [30].

### 2.2. DNA Extraction, Amplification, Sequencing, and Phylogenetic Analysis

In order to obtain 28S rDNA and sequences, the specimens were dried on ethanol in a dry block heater for 1.5 h at 35 °C, digested with a mixture of 49 μL 0.1% Chelex-100 and 1 μL Proteinase K (concentration 10 mg/mL), and incubated for 1 h at 55 °C and 25 min at 95 °C. After that, the water solution of the total DNA was placed into a sterile 500 μL tube and frozen. All DNA was extracted from single worms.

The D1-D3 domain of LSU rDNA (approximately 1000–1300 bp long) was amplified using several primers. The thermal cycle parameters are shown in Supplementary Table S1. The newly obtained sequences from both forward and reverse primers were assembled using Chromas Pro 1.7.4. (Technelysium Pty Ltd., Brisbane, Australia). After assembling and trimming low-quality parts of contigs, the sequences were mounted in general alignment. All specimens were used for PCR after the preliminary morphological examination.

Sequences for general alignment were downloaded from the GenBank database with a custom script based on the “ape” package in the “R studio” [51,52]. Newly obtained sequences were aligned together with others using the “Muscle” algorithm as implemented in the “R studio” “msa” package [53,54]. Information on sequences is given in Supplementary Table S2. The alignment was then trimmed manually in SeaView software to a length of approximately 90% of sequences [55]. The final length of alignment was 1260 bp.

The evolutionary model for Maximum likelihood and Bayesian inference analysis was chosen with MrModeltest v. 2.4 [56]. The best-fitted model was GTR + G + I. Maximum likelihood analysis was performed through the Cipres portal [57] with non-parametric bootstrap with 1000 pseudoreplicates. Bayesian analysis was performed using MrBayes 3.2.7 with computational resources provided by Resource Center “Computer Center of SpbU” [58]. Trees were run as two separate chains (default heating parameters) for 15 million generations, by which time they ceased converging. The quality of the chains was estimated using built-in MrBayes tools and additionally with the Tracer 1.6 package [59]. Based on the estimates by Tracer, the first 25,000 generations were discarded for burn-in.

## 3. Results

### 3.1. Molecular Phylogenetic Analysis

**Figure 1:**
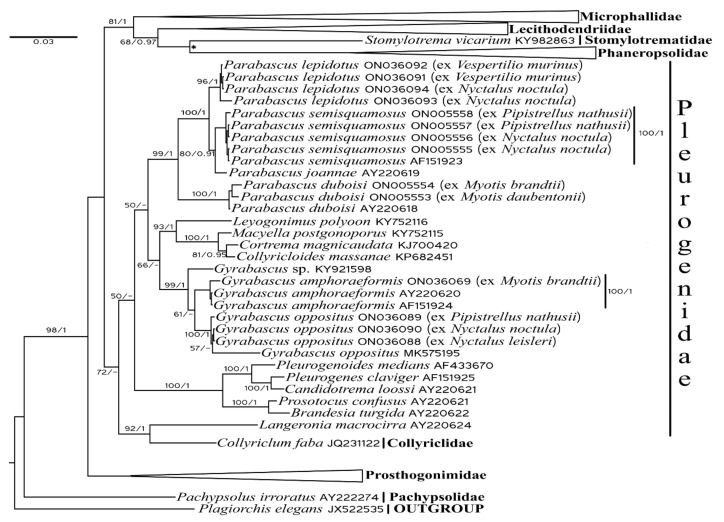
Phylogenetic tree of *Gyrabascus* and *Parabascus* species under consideration based on 28S rRNA gene sequences. Nodal support: ML ≥ 50/BI ≥ 0.9. Node marked with “*” absent in ML analysis.

We generated 14 new sequences of partial 28S rRNA genes for five trematode species and distinguished their relationships with closely related digeneans (Figure 1). We did not discuss the relationships among species of the families Microphallidae, Lecithodendriidae, Phaneropsolidae, and Prosthogonimidae in detail.

In our analysis, Microphallidae is the sister taxa to Lecithodendriidae + Phaneropsolidae + Stomylotrematidae. *Stomylotrema vicarium* (Stomylotrematidae) forms a sister clade to Phaneropsolidae with a relatively low nodal support in Bayesian Inference analysis. In Maximum likelihood analysis, Stomylotrematidae is a separate clade sister to Lecithodendriidae. Pleurogenidae is a sister to the previously described taxa with a relatively low posterior probability. Prosthogonimidae are a basal clade to Microphallidae + Lecithodendriidae + Phaneropsolidae + Stomylotrematidae, and Pleurogenidae. *Pachypsolus irroratus* (Pachypsolidae) is basal to the other trematodes under consideration.

Among pleurogenids, *Gyrabascus* spp. form a sister clade to *Parabascus* spp. with a relatively high Bayesian probability. Three newly generated sequences of *G. oppositus* (ex *N. leisleri*, *N. noctula*, and *P. nathusii*) cluster together with the previously obtained sequence of *G. oppositus* (GenBank No MK575195, ex *P. kuhlii*). *Gyrabascus amphoraephormis* ex *M. brandtii* clustered with other previously obtained specimens of these species. Two specimens of *P. duboisi* (both ex *M. daubentonii* and *M. brandtii*) were found to be closely related to the previously known *P. duboisi* sequence (GenBank No AY220618). Four newly obtained sequences of *P. semisquamosus* (ex *N. noctula* and *P. nathusii*) formed a compact clade without any clear correlation with the host species. *Parabascus joannae* formed a sister clade to *P. semisquamosus*. Four newly obtained specimens of *P. lepidotus* (ex *N. noctula* and *V. murinus*) clustered together without any clear correlation with the host species and form a sister clade to *P. joannae* + *P. semisquamosus*. *Collyriclum* and *Loxogenes* were found to be closely related. Both genera are sister groups to the other pleurogenids.

### 3.2. Systematics and Morphological Characteristics

**Figure 2:**
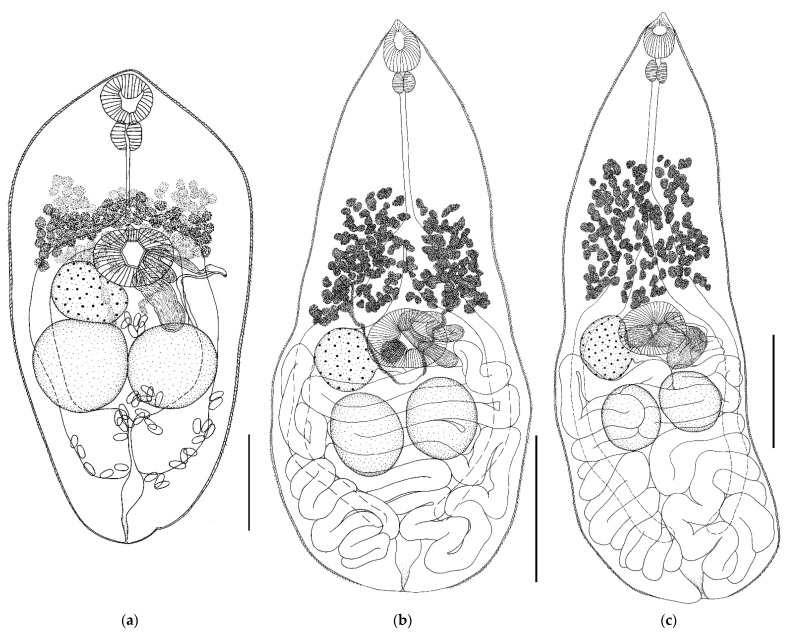
(**a**) *Gyrabascus amphoraeformis* from *Myotis brandtii*, whole view. Scale bar 0.1 mm; (**b**) *Gyrabascus oppositus* from *Pipistrellus nathusii*, whole view. Scale bars 0.2 mm; and (**c**) *Gyrabascus oppositus* from *Nyctalus noctula*, whole view. Scale bar 0.2 mm.

Superfamily Microphalloidea Ward, 1901
Family Pleurogenidae Looss, 1899
Genus *Gyrabascus* Macy 1935
*Gyrabascus amphoraeformis* (Mödlinger, 1930) (Figure 2)
Host: *Myotis brandtii*.
Geographical locality: Smolny village (Republic of Mordovia, Russia).
Availability: GenBank No ON036069.
Accession numbers in collection of IEVB RAS: No 2101.

General description (based on single adult specimen, measurements are given in text directly): Body oval, somewhat narrowed anteriorly and posteriorly; length 494, maximum width 255 at level of intestinal bifurcation. Body length to width ratio 1.9:1. Body covered with spines except at posterior end. Oral sucker subterminal, 53 × 55. Ventral sucker pre-equatorial, transversally elongated, 60 × 85. Oral sucker to ventral sucker width ratio 0.7:1. Pharynx 30 × 32. Prepharynx not visible. Oral sucker to pharynx width ratio 1.7:1. esophagus narrow and long, length 65. Intestinal bifurcation placed before ventral sucker. Intestinal branches end blindly behind testes, not reaching hind body. Testes rounded, post-equatorial; 0.089–0.096 × 0.094–0.099. Cirrus sac absent. Seminal vesicle large, convoluted, located dorsally of ventral sucker. Ejaculatory duct departs from the distal end of seminal vesicle and opens into genital atrium. Genital pore at level of ventral sucker, in the middle between lateral edge of ventral sucker and body margin. Ovary rounded, submedial, located at posterolateral edge of ventral sucker, 62 × 82. Vitellarium consists of numerous irregular-shaped follicles and extends from about mid-esophagus level to anterior ovary edge. Egg size 14–17 × 7–9. Excretory pore terminal.

*Gyrabascus oppositus* (Zdzitowiecki, 1969) (Figure 2)
Host: *Nyctalus noctula*, *Nyctalus leisleri*, *Pipistrellus nathusii*.
Geographical locality: Smolny village (Republic of Mordovia, Russia).
Availability: GenBank No ON036088—ON036090.
Accession numbers in collection of IEVB RAS: No 2102–2105.

General description (based on 25 adult specimens): Body pear-shaped, elongated, with maximum body width at testes level. Body narrows toward conical anterior end of body and widens toward rounded posterior end. Body densely covered with spines except at posterior end. Oral sucker subterminal, drop-shaped, with elongated anterodorsal side. Prepharynx short. Esophagus long. Intestinal bifurcation approximately at border of anterior and middle thirds of body. Intestinal branches long, extending beyond testes level but not reaching posterior body end. Ventral sucker equatorial, transversely elongated, always larger than oral sucker. Testes oval, lying behind ventral sucker in hind body, approximately at the same level or one slightly behind the other. Cirrus sac absent. Seminal vesicle convoluted, lying freely in parenchyma approximately at level of ventral sucker, more or less overlapping with it. Genital pore submedial, opens at ventral sucker level on opposite side from ovary. Ovary round or oval, submedially located at lateral edge of ventral sucker, may be partially overlapped by it. Vitellarium consists of numerous oval or irregularly shaped follicles. Follicles located between level of intestinal bifurcation and ventral sucker, or slightly in front of it. Vitelline fields do not extend below ventral sucker level. Uterus forms numerous transverse loops, occupies all space in hind body, behind level of ventral sucker, and completely overlaps testes. Excretory pore terminal.

*Remarks.* Trematodes *G. oppositus* from *N. noctula* and from *P. nathusii* are morphologically similar and differ in body size only (Table 1 and Figure 2). The body size varied both in specimens from different hosts and in specimens from the same host. Trematodes from *P. nathusii* had the same width as those from *N. noctula* but a smaller body length. So, trematodes ex *P. nathusii* had a body length from 652 to 948 (in *N. noctula* 941–1230) and a width of 348 to 460 (in *N. noctula* 326–450). Correspondingly, the body length to width ratio in trematodes ex *P. nathusii* is 1.8–2.2:1 (average 2.0:1), while for trematodes ex *N. noctula* it is 2.6–3.1:1 (average 2.8:1) (Table 1). Specimens from *P. nathusii* are wider in the posterior half of the body and less elongated, while those from *N. noctula* are more elongated. The size of oral and ventral suckers varied both in specimens from different host species and in specimens from the same host species. Thus, the oral sucker width of trematodes from *N. noctula* varied from 54 to 75, while that of trematodes from *P. nathusii* varied from 49 to 69. The width of the ventral sucker of specimens from *N. noctula* varied from 91 to 114, while that in specimens from *P. nathusii*, from 102 to 126. Accordingly, the oral sucker width of trematodes *G. oppositus* from different hosts ranged from 49 to 75, while the ventral sucker width ranged from 91 up to 126. The width ratio of the oral sucker to ventral sucker remained relatively constant. Larger suckers were noted in specimens from *N. noctula*. The pharynx size varied in *G. oppositus* from the two hosts, being 39–51 in specimens from *N. noctula* and 30–37 in specimens from *P. nathusii* (Table 1). This feature changed only slightly in specimens from one host species. Considerable differences were noted in the esophagus length of trematodes *G. oppositus* both from the two host species and from the same host species. A longer esophagus was noted in *G. oppositus* from *N. noctula*, 148–217 (from *P. nathusii* 126–177) (Table 1). Variability was noted in the size of testes and ovaries in trematodes both from the two host species and from the same host species. The reproductive organs were larger in *G. oppositus* from *N. noctula*. The size of eggs in trematodes varied slightly (Table 1).

**Figure 3:**
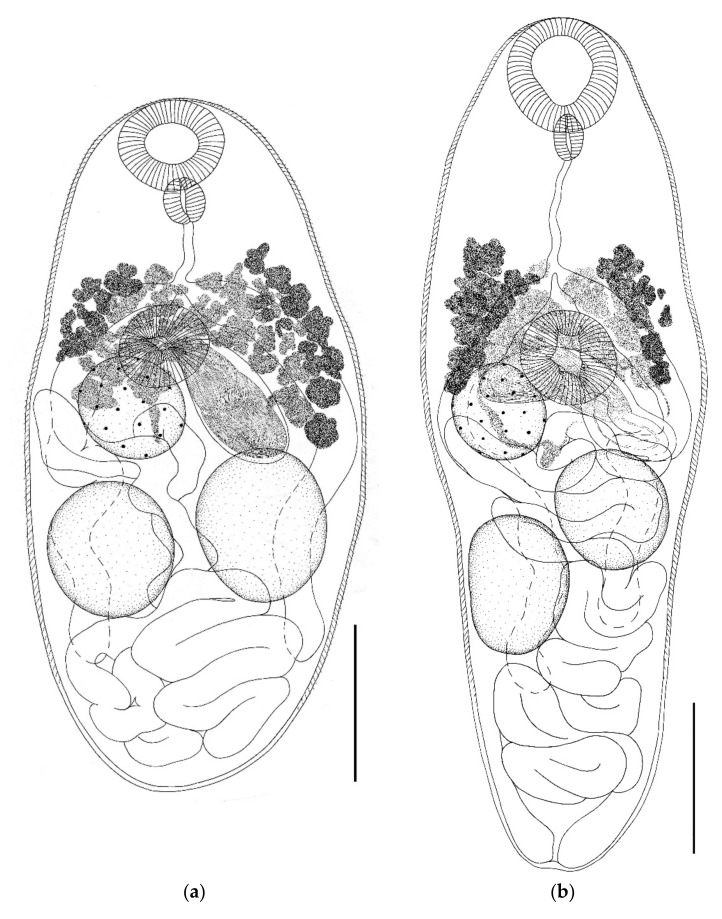

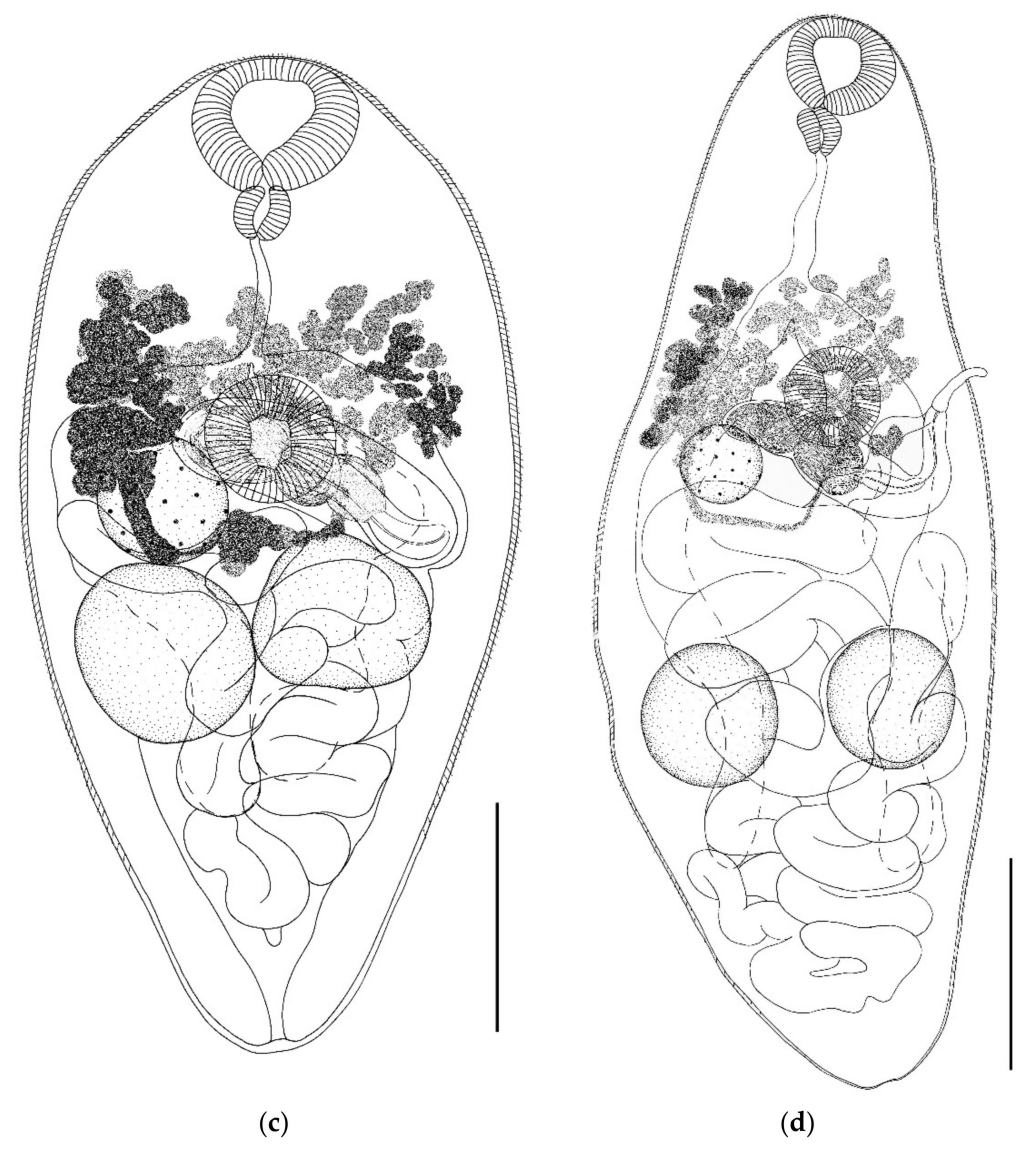
*Parabascus duboisi* from *Myotis daubentonii*, whole views; (**a**–**c**) Scale bar 0.1 mm; (**d**) Scale bar 0.2 mm.

**Figure 4:**
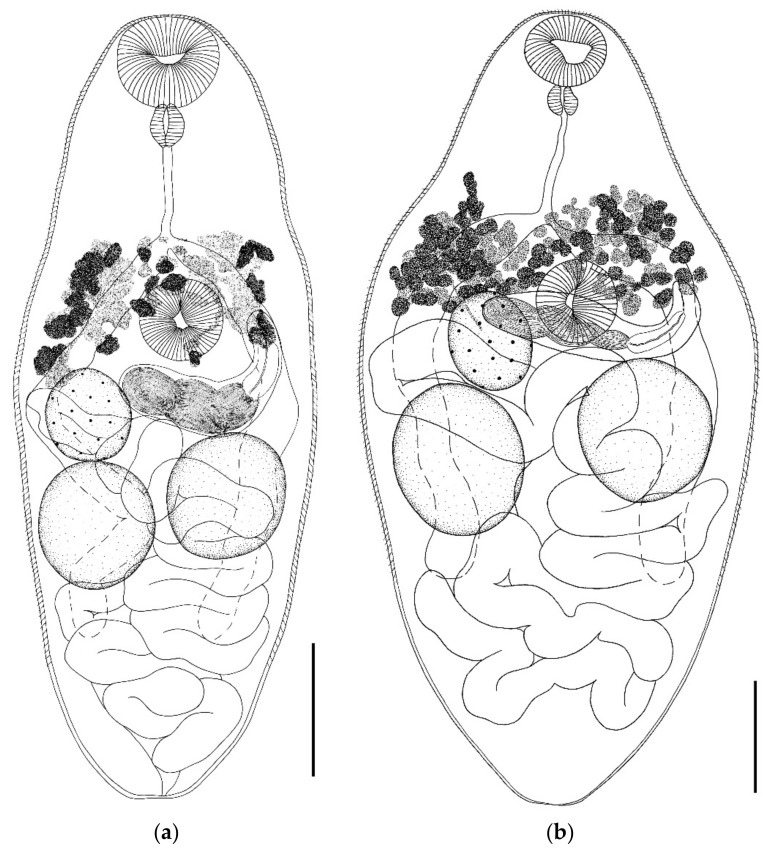

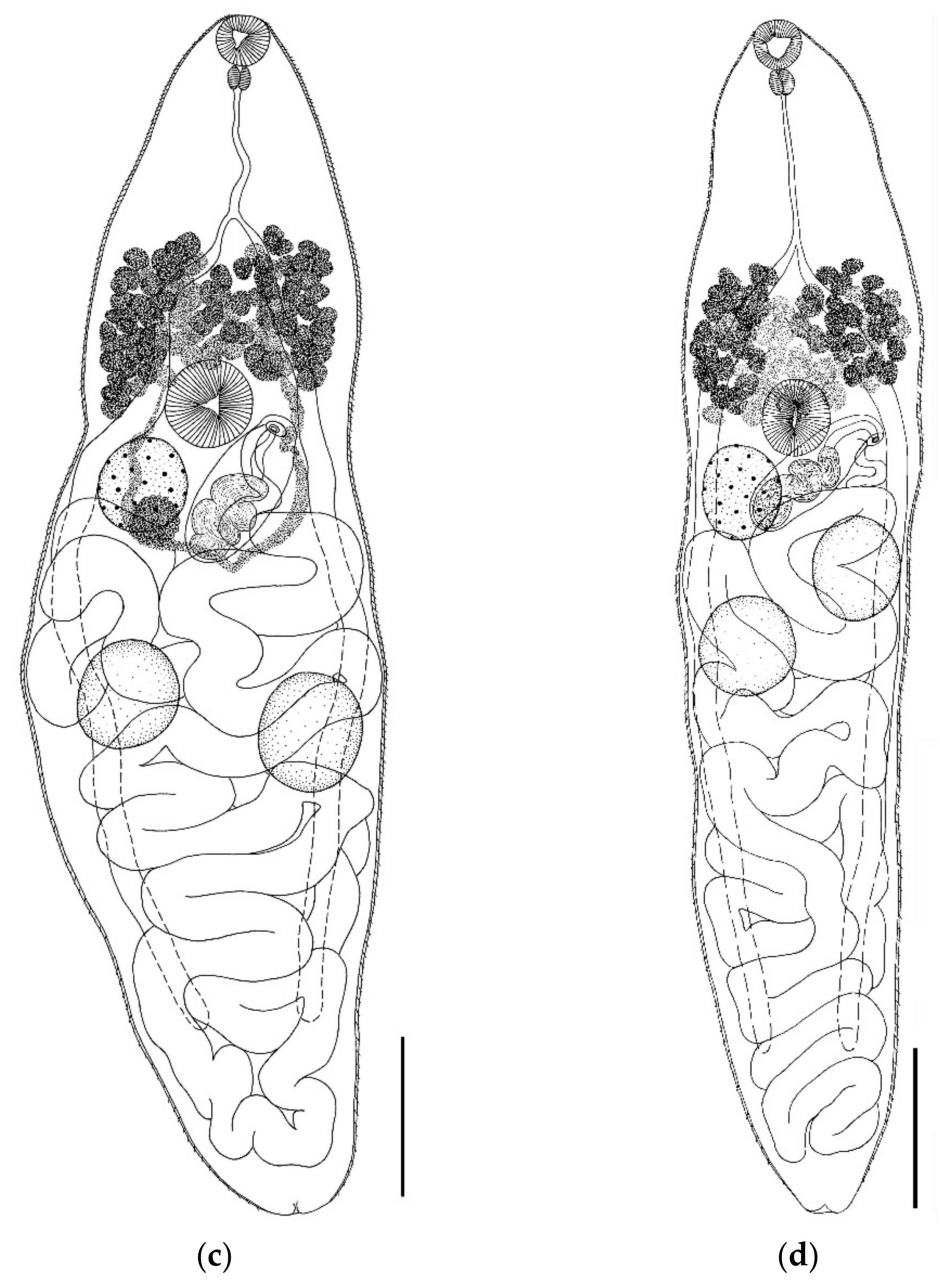
(**a**,**b**) *Parabascus duboisi* from *Myotis brandtii*, whole views; (**c**) *Parabascus semisquamosus* from *Pipistrellus nathusii*; and (**d**) *Parabascus semisquamosus* from *Nyctalus noctula*. Scale bar 0.1 mm.

Genus *Parabascus* Looss, 1907
*Parabascus duboisi* (Hurkova, 1961) (Figure 3 and Figure 4)
Host: *Myotis brandtii*, *Myotis daubentonii*.
Geographical locality: Smolny and Pushta villages (Republic of Mordovia, Russia).
Availability: GenBank No ON005553, ON005554.
Accession numbers in collection of IEVB RAS: No 2106–2108.

General description (based on 28 adult specimens): Body fusiform, ovoid, lanceolate, or oval. Body densely covered with spines except at posterior end. Oral sucker round, subterminal. Prepharynx not visible. Ventral sucker pre-equatorial; equal to or somewhat smaller than oral one. Intestinal bifurcation located at anterior edge of ventral sucker. Intestinal branches long, extending beyond testes level, not reaching hind body. Testes round or oval, located below ovary level or at some distance from it. Testes approximately at same level or one somewhat behind the other. Cirrus sac elongated, club-shaped, located in ventral sucker region or directly behind it, lies across the body, or at an angle to longitudinal axis of body. Proximal part of cirrus sac may touch the ovary, more or less overlap it. Distal part of cirrus sac in most cases reaches the anterior edge of testes or, less often (in three cases), proximal end of cirrus sac touches the testes. Both testes are at some distance from cirrus sac. Convoluted seminal vesicle occupies all proximal parts of cirrus sac. Genital pore submedial at level of ventral sucker or somewhat behind it. Ovary round or oval, located at ventral sucker level or somewhat behind it. Vitellarium consists of numerous irregularly shaped follicles, begins at level of intestinal bifurcation or slightly above it, reaches level of posterior edge of ventral sucker or goes somewhat beyond it. Uterus forms numerous loops, fills all the space below level of ventral sucker, partially overlaps testes. Eggs oval. Excretory pore terminal.

*Remarks.* Specimens of *Parabascus duboisi* from *M. daubentonii* and *M. brandtii* differ in morphology and size of individual organs (Table 1, Figure 3 and Figure 4). The largest specimens of this trematode as well as the smallest ones were noted in *M. daubentonii*. Trematodes from *M. brandtii* were intermediate in size (Table 1). The body length to width ratio varied both in specimens from different hosts and in specimens from one host species. This value varied in trematodes from the two host species in the range from 1.5 to 3.0 (Table 1). The greatest variability in body size was noted in parasites from *M. daubentonii*. Body length varied from 394–1020 (in trematodes from *M. brandtii*, 520–852), and the body width varied from 187–415 (in trematodes from *M. brandtii*, 217–422). The sizes of suckers varied in trematodes regardless of the host species. Thus, the oral sucker width in trematodes from *M. daubentonii* was 63–98 (in trematodes from *M. brandtii* 59–87), while the ventral sucker width in trematodes from *M. daubentonii* was 49–95 (from *M. brandtii* 49–79) (Table 1). The width of the oral sucker to ventral sucker ratio remained relatively constant in specimens from both host species, varying within 1.0–1.4: 1. The pharynx size and the width of the oral sucker to pharynx ratio in *P. duboisi* differed insignificantly. A greater variability was observed in trematodes from one host and even from one host specimen. This was also noted in the case of the length of the esophagus and ovary size. The variability in the size of testes in trematodes from different host species was more marked. The largest testes were noted in trematodes from *M. brandtii*. This also applies to the size of the cirrus sac (Table 1). The body shape changed significantly, both in specimens from different host species and in specimens from the same host species. Variability in the position of the cirrus sac and the interposition of the ovary and testes was noted. Testes could be located directly behind the ovary. In this case, one of the testes could touch the ovary, or the testes were located at some distance from it. The variability of the length of vitelline fields was small (Figure 3 and Figure 4). The egg size in *P. duboisi* was constant and did not depend on the host species.

*Parabascus semisquamosus* (Braun, 1900) (Figure 4)
Host: *Nyctalus noctula*, *Pipistrellus nathusii*.
Geographical locality: Smolny and Pushta villages (Republic of Mordovia, Russia).
Availability: GenBank No ON005555—ON005558.
Accession numbers in collection of IEVB RAS: No 2109–2112.

General description (based on 30 adult specimens): Body elongate, tapering at anterior and posterior ends. Body covered with spines, except at posterior end. Oral sucker subterminal. Prepharynx not visible. Esophagus narrow and long. Intestinal bifurcation approximately in middle part of anterior third of body. Intestinal branches long, extending well beyond testes level, but do not reach hind body. Ventral sucker pre-equatorial, larger than oral sucker, located in posterior part of anterior third of body. Testes rounded or oval, some distance behind ventral sucker, approximately at same level or one slightly behind the other. Cirrus sac elongate and club-shaped, situated immediately behind ventral sucker at an angle to longitudinal axis of body. Convoluted seminal vesicle located in proximal part of cirrus sac. Proximal part of cirrus sac located at lateral or posterolateral edge of ovary and may overlap it to a greater or lesser extent. Convoluted seminal vesicle occupies whole proximal part of cirrus sac. Genital pore is submedial at level of ventral sucker on opposite side from ovary. Ovary rounded or oval, located submedially behind ventral sucker. Vitellarium consists of numerous oval, rounded, and pear-shaped follicles located between intestinal bifurcation and ventral sucker. Vitelline fields do not extend beyond level of posterior edge of ventral sucker. Uterus forms numerous loops, occupies all space behind ovary. Terminal part of uterus with well-defined metraterm. Excretory pore terminal.

*Remarks.* The specimens of *P. semisquamosus* from *N. noctula* and *P. nathusii* are morphologically similar (Figure 4) but differ in the size of the body and individual organs. The body size of specimens from different host species varied widely. The largest trematodes were noted in *N. noctula*: body length 1354–1892 with a width of 277–460; the smallest ones were from *P. nathusii*: body length 1031–1523, with a width of 246–415. Parasites from *P. nathusii* had approximately the same width as those from *N. noctula*, with a smaller body length (Table 1). Accordingly, specimens from *P. nathusii* were wider in the mid body, and those from *N. noctula* were narrower. The variability of the body size was less marked in specimens from the same host species. The size of oral and ventral suckers of *P. semisquamosus* from various hosts was approximately the same, as was their ratio. Considerable differences were noted in the size of oral and ventral suckers from the same host. Thus, the oral sucker width in trematodes from *N. noctula* was 46–67 (from *P. nathusii* 40–68), and the ventral sucker width was 75–106 (from *P. nathusii* 73–110). The same applies to the size of the pharynx (Table 1). The esophagus length of *P. semisquamosus* varied in specimens from the two host species. A longer esophagus was noted in specimens from *N. noctula*: 187–335, as compared with 150–268 in specimens from *P. nathusii*. High variability was observed in the size of reproductive organs of trematodes, regardless of the host species. Larger testes, cirrus sac, and ovary were noted in trematodes from *N. noctula*. Egg size was a constant characteristic in trematodes from both host species.

**Figure 5:**
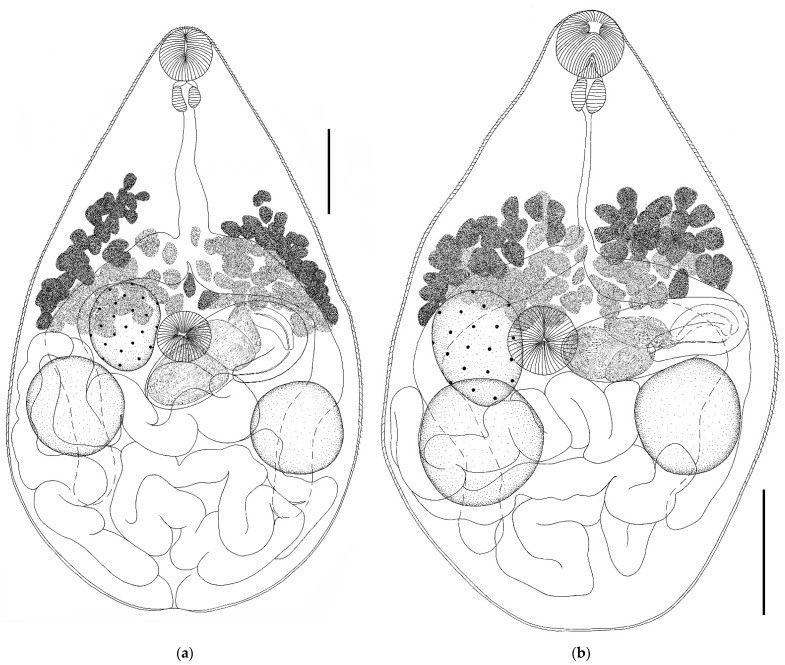

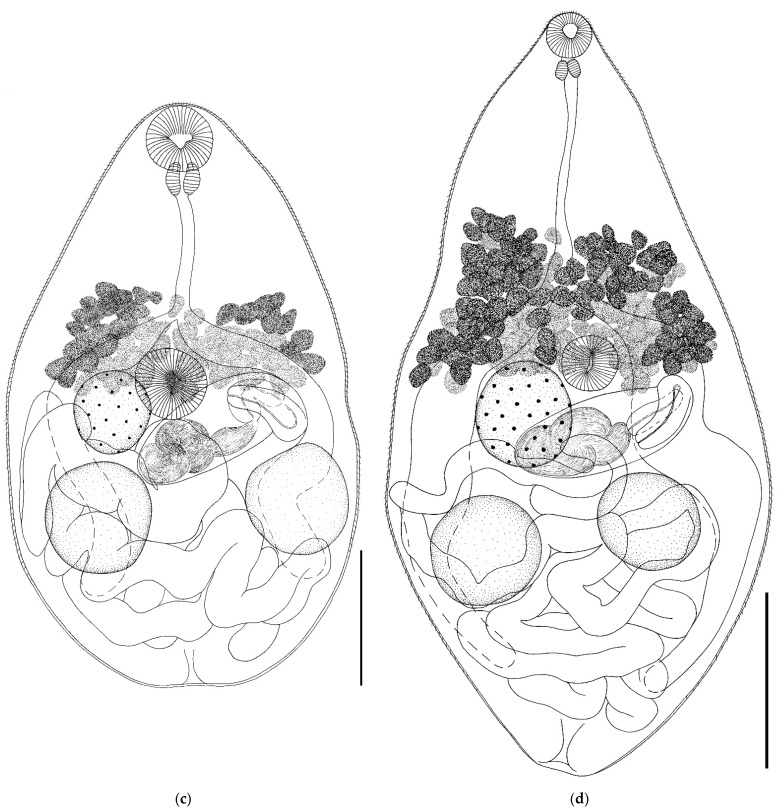
(**a**–**c**) *Parabascus lepidotus* from *Vespertilio murinus*, whole views. Scale bar 0.1 mm; (**d**) *Parabascus lepidotus* from *Nyctalus noctula*. Scale bar 0.2 mm.

*Parabascus lepidotus* Looss, 1907 (Figure 5)
Host: *Nyctalus noctula*, *Vespertilio murinus*.
Geographical locality: Smolny and Pushta villages (Republic of Mordovia, Russia).
Availability: GenBank No ON036091—ON036094.
Accession numbers in collection of IEVB RAS: No 2113–2119.

General description (based on 25 adult specimens): Body pear-shaped or spindle-shaped, covered with spines, except at posterior end. Round or oval oral sucker subterminal. Prepharynx very short and not always visible. Esophagus long. Ventral sucker equatorial, somewhat larger than oral sucker or equal to it. Intestinal bifurcation close to ventral sucker. Intestinal branches long, go beyond testes level, do not reach posterior body end. Testes rounded, located one somewhat behind the other or symmetrically. Cirrus sac elongated, located in ventral sucker region and partially overlapped by it. Convoluted seminal vesicle located in proximal part of cirrus sac. Genital pore submedial, at level of ventral sucker. Ovary round to oval, located at ventral sucker level or somewhat behind it. Vitellarium composed of numerous, irregularly shaped follicles; begins at level below mid-esophagus or intestinal bifurcation, does not go beyond the level of posterior edge of ventral sucker. Uterus forms numerous loops and fills all space in hind body behind the ventral sucker, partially overlapping the testes. Excretory pore terminal.

*Remarks.* Significant variability in size and morphology was noted in *P. lepidotus* from *V. murinus* and *N. noctula* (Figure 5). The largest specimens were noted in *N. noctula*: body length 844–1160 with a width of 385–619; the smallest one, in *V. murinus*: body length 441–770 with a width of 272–519. The variability in body size was especially pronounced in *P. lepidotus* from *V. murinus*. In this host, trematode specimens strikingly different in width were noted. The body length to width ratio in specimens ex *V. murinus* was 1.1–1.8:1 (average 1.6:1), and that in specimens ex *N. noctula* was 1.5–2.5:1 (average 2.1:1). (Table 1). The size of the oral and ventral suckers from the two host species varied. The oral sucker width of specimens from *N. noctula* varied from 51 to 83, while that of specimens from *V. murinus* varied from 43 to 67. The width of the ventral sucker of specimens from *N. noctula* varied from 63 to 98, and that in specimens from *V. murinus*, from 45 to 75. In *P. lepidotus* from *V. murinus*, the oral sucker could be smaller than the ventral sucker, but more often the suckers were equal in size. In trematodes from *N. noctula*, the oral sucker was always smaller than the ventral one. The oral sucker to ventral sucker ratio in specimens from different hosts was constant. This ratio was more variable in trematodes from the same host species. The pharynx of *P. lepidotus* specimens from *N. noctula* was slightly larger than that of the specimens from *V. murinus*. The width of the oral sucker to pharynx ratio was approximately the same in trematodes from various hosts. Considerable variability in the esophagus length was noted in *P. lepidotus* specimens from both host species. The esophagus of specimens from *N. noctula* (217–293) was much longer than that of specimens from *V. murinus* (102–181). The size of the testes, ovary, and cirrus sac varied greatly in *P. lepidotus* specimens from the two host species. These features were less variable in trematodes from the same host species. The largest organs were noted in specimens from *N. noctula*. Differences in the body shape of parasites from different host species were noted. In *P. lepidotus* specimens ex *V. murinus*, the body is pear-shaped with a tapering anterior end and a rounded, widened posterior one. In specimens from *N. noctula*, the body is more spindle-shaped with tapering anterior and posterior ends. Differences in the location of the ventral sucker were also noted. The position of the cirrus sac was different in specimens from various host species. In *P. lepidotus* specimens from *V. murinus*, the cirrus sac was always located submedially at the ventral sucker level, partly overlapped by it. In specimens from *N. noctula*, the cirrus sac was located medially and directly behind ventral sucker. Variability was noted in the length of vitelline fields in specimens from various hosts. In *P. lepidotus* specimens from *V. murinus*, the posterior edge of the vitelline follicles did not extend beyond the ventral sucker level. In *P. lepidotus* specimens from *N. noctula*, the vitelline fields sometimes extended beyond the level of the lower edge of the ventral sucker. The anterior edge of the vitelline fields in *P. lepidotus* specimens from both host species was at the level of intestinal bifurcation or slightly above it. Egg size was constant regardless of the host species.

## 4. Discussion

In this study, we presented morphological descriptions of five species of trematodes from the genera *Gyrabascus* and *Parabascus* from various species of bats from Mordovia (Russia) and novel molecular phylogenetic data on these parasites. The combined use of molecular and morphological methods made it possible to perform a reliable identification of *Gyrabascus amphoraeformis*, *Gyrabascus oppositus*, *Parabascus duboisi*, *Parabascus lepidotus*, and *Parabascus semisquamosus*.

The general topology of the tree obtained in this study is in good agreement with previous publications [30,60,61]. The two exceptions are the families Stomylotrematidae and Phaneropsolidae. In Shchenkov et al. [60] and Sokolov et al. [30], Stomylotrematidae was the closest to Lecithodendriidae, while Phaneropsolidae formed a sister clade to Lecithodendriidae + Stomylotrematidae branch. In Dellagnola et al. [62], Stomylotrematidae and Phaneropsolidae were sister clades to each other and close to Microphallidae. In Dellagnola et al. [62], Lecithodendriidae appeared to be the basal clade to other microphallids. In Fernandes et al. [61], Phaneropsolidae is a sister clade to Lecithodendriidae, while Stomylotrematidae was left out of the analysis. In our analysis, the phylogenetic position of Stomylotrematidae was unstable between ML and BI analyses. All digenean specimens incorporated into our molecular phylogenetic analysis belonged to five distinct species, which were difficult to distinguish based on morphological features only. No impact of host species on the clusterization of the species under consideration was revealed.

In general, the trematode specimens examined in this study corresponded to the earlier morphological descriptions of the species under consideration [17,24,27,28,29,30,33,35,36,37,38,39,47,48,63,64,65,66,67,68]. However, we noted some differences in the topology of the inner organs and the morphometric features. Specimens of *Gyrabascus* spp. and *Parabascus* spp. from various host species and from specimens of the same host showed a broad morphometric variability. Morphometric features such as the body length and width, and the size of the reproductive organs varied broadly, apparently depending to a large extent on the age of the parasites. The dependence of morphometric changes on the trematode age has been noted by several authors [69,70,71,72,73,74] and is especially evident in trematodes from the same host species [73].

Morphological variability of the body shape and the position of the cirrus sac noted in our study also depended on the degree of development of the trematode specimen. The variability of the length of the vitelline fields, the body length to width ratio, and the oral sucker to pharynx ratio were less pronounced. Features such as the oral sucker to the ventral sucker width ratio and the size of eggs were relatively stable (Table 1).

The observation that the variability of parasites depends on the host species has been made in a number of studies [70,71,73,74,75,76,77,78,79]. We also found that the morphological variability of trematodes depended on the species of the host. A host-induced variability was recorded in *P. oppositus*, *P. duboisi*, *P. semisquamosus*, and *P. lepidotus* in respect of the body size, body shape, and the size of inner organs (Table 1 and Figure 2, Figure 3, Figure 4 and Figure 5).

We gave the first complete description of *G. amphoraeformis* from *M. brandtii*, including morphometric data. The comparison of our specimen of *G. amphoraeformis* with the descriptions of other authors [27,28,33,36,38,48,63,64,65,67] demonstrated a good agreement in the main morphological and morphometric characteristics, except for the egg size. In our specimen, the eggs were somewhat smaller than in descriptions by other authors (Section 3.2 and Table 2).

The specimens of *G. oppositus* described by us ex *N. noctula* differed from the previously described specimens from other host species in being longer (Table 3). It should be noted that this species has been previously recorded in *P. kuhlii*, *M. schreibersi*, and *Eptesicus serotinus* Schreber, 1774 [28,30,39]. *Pipistrellus nathusii* and *N. noctula* are new hosts for *G. oppositus*.

Specimens of *P. semisquamosus* examined in our study fully corresponded to the descriptions available in the literature [17,24,27,28,29,33,34,64,80]. Specimens of *P. duboisi* examined here differed in body length, size of the oral sucker, and cirrus sac (Table 1 and Table 4) from those described by other authors [17,24,27,28,29,63,66,67]. The difference was mostly due to the fact that our specimens from *M. daubentonii* were relatively larger than the parasites described earlier. Our specimens of *P. duboisi* from *M. brandtii* fully corresponded to the literature data in respect of the measurements (Table 4).

The specimens of *P. lepidotus* from *V. murinus* obtained in our study were smaller in respect of body size than those described in earlier studies [17,24,28,33,35,37,38,47,63]. Accordingly, their organs were also smaller (Table 1). Specimens of *P. lepidotus* from the *N. noctula* mainly corresponded to the morphological and morphometric characteristics given earlier (Table 5).

Related species of trematodes can be identified erroneously, therefore, it is necessary to take into account the morphological and morphometric variability in their diagnosis. Zdzitowiecki [28] noted that *P. duboisi* and *P. lepidotus* are morphologically very similar. The results of our study confirm this. It was often difficult to distinguish these species. For example, such a diagnostic morphometric feature as the size of the oral and the ventral sucker was not always applicable. In our material, there were trematode specimens (both *P. duboisi* and *P. lepidotus*) in which the size of the oral sucker was equal to that of the ventral sucker. Another diagnostic feature that could not be used in these two species was the esophagus length. There were specimens in which the values of this feature were the same. We conclude that the following morphological and morphometric features should be taken into account when identifying these species: the body length to width ratio, the oral sucker to pharynx width ratio, the oral sucker to the ventral sucker width ratio, and the location of the ventral sucker relative to the mid body.

Zdzitowiecki [28] has also noted that *G. oppositus* is similar in many morphological features to *P. lepidotus* and that many early descriptions of the latter [36,37,38,63] could also apply to *G. oppositus*. This is probably why Khotenovsky [29] reduced *G. oppositus* to synonyms of *P. lepidotus*. In addition, these species of trematodes have a common host, *N. noctula*. Therefore, when identifying these trematode species, one should take into account the following morphological features: the body shape, the oral sucker shape, the size and position of the ventral sucker relative to the mid body, and the presence/absence of the cirrus sac.

In turn, *G. oppositus* is similar to *G. amphoraeformis*. Skvortsov [49] considered the former as a synonym of the latter. Distinctive morphological features for these two species are the body shape, the oral sucker shape, the position of the ventral sucker relative to the mid body and the location of the genital pore.

It is much easier to distinguish *P. semisquamosus* from *P. lepidotus* and *P. duboisi*. Here, the distinctive morphological and morphometric features are the body shape, the body length to width ratio, the oral sucker to the ventral sucker width ratio, the position of the ventral sucker relative to the mid body, and the length of the intestinal branches.

Based on our results, we propose a key for identifying *Gyrabascus* spp. and *Parabascus* spp. involved in our study. It should be noted, however, that the applicability of this key is somewhat limited due to a broad morphological variability of these trematodes, which is especially pronounced in immature specimens. Special care should also be taken during the identification of adults that could have been deformed during fixation and whole mount preparation.

1(6) Cirrus sac absent.
2(4) Genital pore marginal.
3(2) Body oval with a maximum width at the intestinal bifurcation level. Ventral sucker pre-equatorial ...................... *Gyrabascus amphoraeformis* (Mödlinger, 1930).
4(2) Genital pore submedial.
5(4) Body pear-shaped with a maximum width at the testes level. Ventral sucker equatorial ………….………………….... *Gyrabascus oppositus* (Zdzitowiecki 1969).
6(1) Cirrus sac present.
7(12) Ventral sucker pre-equatorial.
8(10) Ventral sucker larger than oral sucker.
9(8) Body elongated, narrow. Intestinal branches extend far beyond the testes level. The body length to width ratio is 3.2–5.2: 1. The oral sucker to ventral sucker ratio is 0.6–0.7:1. The oral sucker to pharynx ratio is 1.6–2.3:1 ...................
  ...................................................................... *Parabascus semisquamosus* (Braun, 1900).
10(8) Ventral sucker equal to or less than oral sucker.
11(10) Body oval, shortened. Intestinal branches end directly behind the testes. The body length to width ratio is 1.5–3.0:1. The oral sucker to ventral sucker ratio is 1.0–1.4:1. The oral sucker to pharynx ratio is 2.0–3.0:1 ……………….…..……..
  ………………………………………………….. *Parabascus duboisi* (Hurkova, 1961).
12(7) Ventral sucker equatorial.
13(12) Body pear-shaped with a maximum width at the testes level. The body length to width ratio is 1.1–2.5:1. The oral sucker to ventral sucker ratio is 0.7–0.9:1. The oral sucker to pharynx ratio is 1.6–2.3:1 ....... *Parabascus lepidotus* (Looss, 1907).

An analysis of the literature data and the results of our own studies showed that trematodes of the genera *Gyrabascus* and *Parabascus* exhibit a certain host specificity. In our study, *P. lepidotus* was found only in *N. noctula* and *V. murinus*. *P. semisquamosus* and *G. oppositus* were noted only in *P. nathusii*, *N. noctule*, and *N. leisleri*. *Parabascus duboisi* was recorded only in *Myotis* spp. We found two specimens of *G. amphoraeformis* in *M. brandtii*.

*Gyrabascus amphoraeformis* mainly occurs in *Myotis* bats. Though several authors reported this species from *N. noctula*, *P. pipistrellus*, *E. serotinus*, and *Barbastella barbastellus* Schreber, 1774 [24,28,33], all of them provided drawings of specimens from *Myotis* spp., and so these reports should be treated with caution. However, the presence of *G. amphoraeformis* in *P. kuhlii* has been confirmed by molecular analysis [40], indicating that *G. amphoraeformis* may indeed parasitize bats other than *Myotis* spp.

*Gyrabascus oppositus* has been previously noted only by Mituch [39] (as *P. semisquamosus*), Zdzietowiecki [28], and Sokolov et al. [30] from *P. kuhlii*, *M. schreibersii*, and *E. serotinus*. In this study, we added three new species to the list of hosts of *G. oppositus*.

According to the literature data, the hosts of *P. semisquamosus* are *N. noctula* and *P. pipistrellus* (Table 6). Khotenovsky [29] is the only source where *M. daubentonii* is indicated as its host. The description and drawing of the parasite in this work match those of *P. semisquamosus*, but we believe that an error could have occurred in identifying the host. Sharpilo and Iskova [33] report *P. semisquamosus* from *Myotis mystacinus* Kuhl, 1817 (the drawing and description of the specimen from *N. noctula*). Skvortsov [24] doubts the findings of *P. semisquamosus* in *Myotis* bats since the trematode is a specific parasite of *Nyctalus* and *Pipistrellus* bats. We have never found *P. semisquamosus* in *Myotis* spp. in our long-term studies of bat helminths [15,16,17,18,19,20,21,22,23].

*Parabascus lepidotus* has previously been recorded mainly in *E. serotinus*. Other bats mentioned as its hosts are *E. nilssoni*, *N. noctula*, *P. kuhlii*, *P. pipistrellus*, *P. auritus*, *V. murinus*, *M. blythii*, and *M. nattereri* [17,24,28,29,33,35,37,38,47,63]. Findings of *P. lepidotus* in *Myotis* bats require confirmation, as the authors may have dealt with a closely related species, *P. duboisi*, which is a common parasite of *Myotis* spp. No drawings of *P. lepidotus* from *Myotis* bats are given in the studies cited above, except for Zdzietowiecki [28]. His work contains a drawing of a parasite from *M. nattereri*, which, though we cannot be certain, seems very similar to *P. duboisi*. We have critically reviewed whole mounts of trematodes from bats of the Samarskaya Luka (Russia) [16,17] and found that of all *Parabascus* spp. only *P. duboisi* parasitizes Myotis bats.

*Parabascusduboisi* is a specific parasite of *Myotis* spp. [17,24,27,28,29,63,66,67]. Morozov [66] notes *M. daubentonii* and *P. pipistrellus* as hosts of *P. semisquamosus*, but the description and the drawing of the trematodes from *M. daubentonii* match that of *P. duboisi*. The trematodes from *P. pipistrellus* were correctly identified as *P. semisquamosus*. Revision of whole mounts of trematodes from bats of the Samarskaya Luka showed that *E. nilssoni* is parasitized by *P. lepidotus* [16,17].

## 5. Conclusions

The combined use of molecular and morphological methods in our study made it possible to reliably identify closely related trematode species *Gyrabascus amphoraeformis*, *Gyrabascus oppositus*, *Parabascus duboisi*, *Parabascus lepidotus*, and *Parabascus semisquamosus*. A broad morphological variability of *Gyrabascus* spp. and *Parabascus* spp. was revealed, both from various host species and from various specimens of the same host species. We reevaluated morphological characters for a reliable identification of the closely related species of the genera *Gyrabascus* and *Parabascus* involved in our study and proposed a key for their identification.

Our data complement and expand the knowledge of bat parasites. We provided the first record of *Gyrabascus amphoraeformis* from bats in the Volga basin and the first record of *Gyrabascus oppositus* from bats in the Middle Volga region. We also established three new hosts of *Gyrabascus oppositus*: *N. leisleri*, *N. noctula*, and *P. nathusii*.

## Figures and Tables

**Table 1:** Measurements of trematode species in our study.

	*Gyrabascus oppositus*		*Parabascus duboisi*		*Parabascus semisquamosus*		*Parabascus lepidotus*	
Host	*N. noctula*	*P. nathusii*	*M. daubentonii*	*M. brandtii*	*P. nathusii*	*N. noctula*	*V. murinus*	*N. noctula*
Locality	Smolny	Smolny	Pushta	Smolny	Smolny	Pushta	Pushta	Pushta
Body length	941–1230 (1073)	652–948 (776)	394–1020 (611)	520–852 (679)	1031–1523 (1343)	1354–1892 (1608)	441–770 (599)	844–1160 (967)
Body width	326–450 (382)	348–460 (394)	187–415 (245)	217–422 (348)	246–415 (362)	277–460 (354)	272–519 (386)	385–619 (479)
OS length	79–110 (94)	69–91 (81)	56–95 (68)	55–79 (67)	40–63 (53)	47–63 (55)	43–63 (52)	49–79 (64)
OS width	54–75 (67)	49–69 (60)	63–98 (74)	59–87 (75)	40–68 (54)	49–67 (59)	43–67 (54)	51–83 (68)
VS length	91–114 (104)	102–126 (110)	49–95 (64)	49–79 (67)	73–110 (90)	75–106 (92)	45–75 (60)	63–98 (77)
VS width	110–138 (126)	79–106 (91)	51–91 (64)	47–79 (66)	79–113 (93)	75–110 (98)	43–69 (58)	55–106 (78)
Pharynx length	39–51 (44)	30–37 (33)	24–39 (31)	24–35 (29)	24–39 (31)	30–39 (34)	24–32 (27)	24–43 (32)
Pharynx width	39–51 (45)	30–37 (34)	24–39 (31)	22–35 (31)	24–39 (31)	26–41 (33)	24–34 (29)	24–45 (37)
Esophagus	148–217 (178)	126–177 (156)	40–122 (71)	51–118 (87)	150–268 (189)	187–335 (268)	102–181 (135)	217–293 (240)
Testes length	100–158 (125)	98–126 (114)	63–138 (101)	87–146 (114)	118–165 (135)	118–193 (151)	71–130 (106)	106–185 (143)
Testes width	91–130 (109)	86–106 (96)	67–118 (86)	87–138 (106)	87–134 (115)	91–165 (126)	75–118 (103)	106–169 (133)
Cirrus sac length	–	–	158–275 (198)	197–276 (234)	138–226 (153)	177–267 (215)	154–205 (175)	191–311 (248)
Cirrus sac width	–	–	34–67 (46)	37–55 (45)	51–93 (60)	59–110 (80)	51–79 (64)	67–85 (78)
Ovary length	110–138 (120)	86–118 (97)	47–98 (66)	51–102 (75)	87–146 (113)	96–157 (126)	60–102 (91)	102–165 (126)
Ovary width	87–114 (103)	78–110 (91)	47–106 (68)	55–98 (76)	79–134 (102)	83–139 (114)	55–98 (82)	104–165 (117)
Eggs length	24–30 (27)	23–26 (25)	20–26 (23)	20–26 (24)	19–24 (21)	19–24 (21)	19–24 (20)	20–24 (21)
Eggs width	12–16 (14)	11–14 (12)	12–16 (13)	12–16 (14)	8–12 (10)	8–12 (10)	10–14 (12)	11–14 (12)
Body length/width ratio	2.6–3.1 (2.8)	1.8–2.2 (2.0)	1.5–3.0 (2.5)	1.6–2.8 (2.0)	3.2–4.9 (3.7)	3.3–5.2 (4.6)	1.1–1.8 (1.6)	1.5–2.5 (2.1)
OS/VS width ratio	0.5–0.6 (0.5)	0.5–0.6 (0.6)	1.0–1.4 (1.2)	1.0–1.2 (1.1)	0.6–0.7 (0.6)	0.6–0.7 (0.7)	0.7–0.9 (0.8)	0.7–0.9 (0.8)
OS/pharynx width ratio	1.3–1.7 (1.5)	1.5–1.9 (1.8)	2.0–2.8 (2.5)	2.1–3.0 (2.4)	1.6–1.9 (1.7)	1.6–2.3 (1.8)	1.7–2.1 (1.9)	1.6–2.3 (1.8)

Note: Here and in other tables, measurements in micrometers. Mean values are given in parentheses; OS—oral sucker, VS—ventral sucker.

**Table 2:** Measurements of *Gyrabascus amphoraeformis* from the original description and redescriptions.

	Modlinger [38]	Dubois [64,65]	Hurkova [37,63]	Odening [27]	Zdzietowiecki [28]	Matskasi [67]	Khotenovsky [48]	Sharpilo, Iskova [33]
Host	*M. blythii*	*M. mystacinus*, *M. daubentonii*	*M. mystacinus*, *M. daubentonii*, *M. emarginatus*, *M. myotis*, *P. austriacus*	*M. myotis*	*M. myotis*, *M. dasycneme*, *M. daubentonii*, *M. mystacinus*, *B. barbastellus*	*M. daubentonii*	*M. daubentonii*	*M. bechsteinii*, *M. myotis*, *N. noctula*, *E. serotinus*
Locality	Hungary	France, Switzerland	former Czechoslovakia	Germany	Poland	Hungary	Russia	Ukraine
Body size	580–590 × 390	600–720 × 370–540	370–770 × 270–610	1050–1210 × 650–820	351–865 × 207–572	555 × 475	420–630 × 240–340	820–950 × 550–680
Oral sucker	50–60	60–90	40–70	80–90	39–94 × 44–84	43×50	40–70	66–88 × 77–93
Ventral sucker	140 ^1^	100–200 × 120–160	80 × 140	120–170 × 140–180	63–129 × 80–173	95 × 125	60–80 × 80–100	140–150 × 130–150
Pharynx	50–60	40–50	30–40	40–50 × 500–600	23–55 × 26–58	–	30–50	33–44 × 38–49
Esophagus	Very short	60–100	–	140–230	44–171	–	50–150	110–160
Testes	260–270 × 180–190	100–160 × 160–200	110–200 × 80–140	260–340 × 150–210	58–182 × 46–202	125×87	60–120 × 70–110	170–270 × 140–170
Ovary	130 ^1^	60–100 × 90–130	80–140 × 60–90	110–220×100–230	43–110 × 44–110	80 × 65	60–80 ^1^	0.11–0.16 × 0.11–0.15
Eggs	26 × 11	21–26 × 10–12	20–26 × 8–13	19–23 × 9–12	21–27 × 10–14	–	20–28 × 11–14	20–22 × 10–13

Note: ^1^—diameter.

**Table 3:** Measurements of *Gyrabascus oppositus* from the original description and redescriptions.

	Sokolov et al. [30]	Mituch [39]	Zdzietowiecki [28]
Host	*Pipistrellus kuhlii*	*Miniopterus schreibersii*	*Eptesicus serotinus*
Locality	Russia	former Czechoslovakia	Poland
Body size	620–1010 × 330–440	1077 × 385	763–1070 × 370–547
Oral sucker	89–108 × 57–70	93 × 66	63–72 × 71–73
Ventral sucker	82–108 × 108–133	146 × 106	103–121 × 137–148
Pharynx	38–51 × 38–51	–	42–48 × 39–49
Esophagus	120–215	266	125–178
Testes	90–152 × 76–138	106 × 66	94–158 × 90–142
Ovary	82–114 × 95–152	100 × 60	69–105 × 77–87
Eggs	24–26 × 13–16	15 × 9	24–28 × 12–14

**Table 4:** Measurements of *Parabascus duboisi* from the original description and redescriptions.

	Morozov [66]	Hurkova [63]	Odening [27]	Zdzietowiecki [28]	Skvortsov [24]	Matskasi [67]	Khotenovsky [29]	Kirillov et al. [17]
Host	*M. daubentonii*, *P. pipistrellus*	*M. dasycneme*, *M. dasycneme*, *M. mystacinus*	*M. daubentonii*, *M. myotis*	*M. daubentonii*, *M. dasycneme*, *M. emarginatus*	*M. daubentonii*	*M. daubentonii*	*M. daubentonii*	*M. dasycneme*
Locality	Belarus	former Czechoslovakia	Germany	former Czechoslovakia, Poland	Moldova	Hungary	Russia	Russia
Body size	700–820 × 350–460	550–910 × 220–370	323–595 × 198–330	478–984 × 191–432	292–986 × 132–520	950 × 580	length 290–900	667–815 × 274–414
Oral sucker	70–80 × 60–90	50–80 ^1^	49–62 × 42–72	54–86 × 65–94	42–74 × 45–93	62 × 75	53–100 ^1^	74–82 × 78–85
Ventral sucker	70 × 60–70	50–70 ^1^	40–60 × 46–58	54–78 × 56–87	39–98 × 42–101	63 × 100	46–90 ^1^	67–76 × 74–79
Pharynx	30–40 ^1^	20–40 ^1^	23–26 × 21–33	24–43 × 26–43	21–39 × 26–42	–	22–24 ^1^	24–39 × 28–43
Esophagus	80–110	60–100	40–99	52–125	34–133	–	–	52–98
Testes	100–170 × 80–140	80–130 × 80–140	67–107 × 56–90	69–161 × 64–142	58–172 × 79–207	150 × 125	–	72–93 × 76–122
Cirrus sac	190–260 × 80–140	120–220 × 50–100	86–151 × 28–51	38–81	86–255 × 42–79	–	Small	143–212 × 47–58
Ovary	90–110 × 80–140	60–80 × 60–100	37–81 × 33–74	54–116 × 48–119	53–138 × 58–159	90 × 130	Spherical	62–86 × 71–94
Eggs	22–25 × 11–13	22–26 × 12–16	19–23 × 9–12	21–26 × 12–15	21–26 × 18–21	–	22–28 × 12–16	18–24 × 8–12

Note: ^1^—diameter.

**Table 5:** Measurements of *Parabascus lepidotus* Looss, 1907 from the original description and redescriptions.

	Looss [35]	Modlinger [38]	Hurkova [37,63]	Zdzietowiecki [28]	Skvortsov [24]	Khotenovsky [47]	Sharpilo, Iskova [33]	Kirillov et al. [17]
Host	*P. kuhlii*	*E. serotinus*	*N. noctula*, *P. pipistrellus*	*E. serotinus*, *M. nattereri*	*E. serotinus*	*E. serotinus*	*E. serotinus*	*E. nilssoni*
Locality	Egypt	Hungary	former Czechoslovakia	Poland	Moldova	Uzbekistan	Ukraine	Russia
Body size	1100 × 430	860 × 350	1030–1300 × 460–650	836–847 × 366–424	854–1396 × 530–686	790–1300 × 360–600	1000–1400 × 670–780	907–1277 × 461–675
Oral sucker	80 ^1^	70 ^1^	70–80 ^1^	66–68 × 56–77	69–93 × 79–98	60–90 ^1^	70–75 × 82–115	63–85 × 78–91
Ventral sucker	150 ^1^	150 ^1^	130–140 ^1^	82–101 × 74–94	96–127 × 111–148	100–150 ^1^	105–110 × 90–120	83–94 × 86–98
Pharynx	60 × 30	50 ^1^	40–50 ^1^	32–41 × 28–43	37–53 × 47–58	30–60 ^1^	38–43 ^1^	32–36 × 35–49
Esophagus	Long	Long	220–230	141–170	172–287	110–230	220–270	220–272
Testes	Large	200 ^1^	100–120 × 150–170	117–198 × 101–157	106–178 × 172–226	120–220 × 130–210	140–240 ^1^	114–137 × 128–143
Cirrus sac	–	–	220–230 × 90	Width 110	212–411 × 85–118	200–300 × 80–110	270–300 × 80–120	216–305 × 78–83
Ovary	–	150 ^1^	130–140 × 90–100	86–144 × 75–81	118–145 × 122–178	110–160 ^1^	130–150 × 110–130	98–116 × 102–125
Eggs	22–23 × 13	29–30 × 11	24–25 × 13–15	18–23 × 11–15	18–23 × 10–13	20–25 × 11–14	21–23 × 11–13	20–24 × 11–14

Note: ^1^—diameter.

**Table 6:** Measurements of *Parabascus semisquamosus* from the original description and redescriptions.

	Braun [34]	Soltys [80]	Dubois [64]	Odening [27]	Zdzietowiecki [28]	Skvortsov [24]	Khotenovsky [29]	Sharpilo, Iskova [33]	Kirillov et al. [17]
Host	*N. noctula*	*N. noctula*	*N. noctula*	*P. pipistrellus*	*N. noctula*	*N. noctula*	*M. daubentonii*	*N. noctula*	*P. nathusii*
Locality	Germany	Poland	France	Germany	Poland	Moldova	Russia	Ukraine	Russia
Body size	1500 × 260	1000–1560 × 250–400	1470–1960 × 310–370	1210–1630 × 470–630	1347 × 254	266–1712 × 118–421	900–2600	1300–1800 × 330–470	1061–1617 × 215–446
Oral sucker	70–90 ^1^	50–80 ^1^	70–80 × 80	51–52 × 62–72	63–66 × 65–74	45–74 × 47–82	40–90 ^1^	55–77 × 60–88	47–60 × 55–71
Ventral sucker	110 × 90	–	90–100 × 130–140	106–110 × 109–114	87–107 × 82–105	39–106 × 47–118	52–140 ^1^	93–125 × 99–130	59–88 × 67–90
Pharynx	40 ^1^	30 ^1^	40–50 ^1^	28–32 × 26–38	32–35 × 33–36	21–26 × 31–39	19–38 ^1^	30–40 ^1^	25–31 × 30–35
Esophagus	Medium length	160–240	260	106–131	282	85–250	–	220–360	192–268
Testes	–	150–200	160–200 × 140–170	144–228 × 104–172	103–135 × 73–91	63–140 × 87–174	–	80–190 × 100–170	90–118 × 74–87
Cirrus sac	–	120–200 × 70	310–350 × 90–100	224–259 × 93–104	Width 74–75	106–258 × 47–98	–	220–300 × 60–100	190–200 × 58–79
Ovary	Postacetobular	150–180	160–170 × 130–140	127–135 × 90–143	96–166 × 78–94	61–106 × 54–159	–	65–160 × 66–140	67–83 × 75–98
Eggs	23–18	20 × 10	20–22 × 10–14	18–21 × 9–14	18–22 × 11–13	18–23 × 13–15	18–25 × 9–15	19–22 × 11	17–20 × 9–11

Note: ^1^—diameter.

## Data Availability

GenBank numbers are given in the relevant section of the manuscript. Any other data is available after a reasonable request.

## Supplementary Materials

The following supporting information can be downloaded at: https://www.mdpi.com/article/10.3390/biology11060878/s1, Table S1: PCR conditions used in the present study [40,81,82,83]; Table S2: Sequences used in molecular phylogenetic analysis [30,40,41,42,84,85,86,87,88,89,90,91,92,93,94,95,96,97,98,99,100,101,102,103].
